# Supplementary material for: Epigenetic conflict on a degenerating Y chromosome increases mutational burden in Drosophila males
Source: Nat Commun. 2020 Nov 2;11:5537. doi: 10.1038/s41467-020-19134-9 (PMC7608633; doi:10.1038/s41467-020-19134-9)
Supplement: Supplementary file 3 — Reporting Summary [file 41467_2020_19134_MOESM3_ESM.pdf]

nature research

Corresponding author(s): Bachtrog
Last updated by author(s): Sep 17, 2020

Reporting Summary

Nature Research wishes to improve the reproducibility of the work that we publish. This form provides structure for consistency and transparency in reporting. For further information on Nature Research policies, see our Editorial Policies and the Editorial Policy Checklist.

Statistics

For all statistical analyses, confirm that the following items are present in the figure legend, table legend, main text, or Methods section.

n/a	Confirmed	
☐	☒	The exact sample size (*n*) for each experimental group/condition, given as a discrete number and unit of measurement
☐	☒	A statement on whether measurements were taken from distinct samples or whether the same sample was measured repeatedly
☐	☒	The statistical test(s) used AND whether they are one- or two-sided<br>*Only common tests should be described solely by name; describe more complex techniques in the Methods section.*
☐	☒	A description of all covariates tested
☒	☐	A description of any assumptions or corrections, such as tests of normality and adjustment for multiple comparisons
☐	☒	A full description of the statistical parameters including central tendency (e.g. means) or other basic estimates (e.g. regression coefficient) AND variation (e.g. standard deviation) or associated estimates of uncertainty (e.g. confidence intervals)
☐	☒	For null hypothesis testing, the test statistic (e.g. F, t, r) with confidence intervals, effect sizes, degrees of freedom and P value noted<br>*Give P values as exact values whenever suitable.*
☒	☐	For Bayesian analysis, information on the choice of priors and Markov chain Monte Carlo settings
☒	☐	For hierarchical and complex designs, identification of the appropriate level for tests and full reporting of outcomes
☐	☒	Estimates of effect sizes (e.g. Cohen's *d*, Pearson's *r*), indicating how they were calculated

Our web collection on statistics for biologists contains articles on many of the points above.

Software and code

Policy information about availability of computer code

Data collection	No software was used to collect data.
Data analysis	bwa (r0.7.15)<br>bowtie2 (v2.3.0)<br>bedtools (v2.26.0)<br>samtools (v1.5)<br>RepeatMasker (v3.3)<br>featureCounts (v1.6.2, part of subread package)<br>Rstudio (v1.3)<br>IRanges (v2.22)<br>DEseq2 (v1.28.1)<br>Custom scripts used for analysis are available on KW's Github page: https://github.com/weikevinhc/heterochromatin.

For manuscripts utilizing custom algorithms or software that are central to the research but not yet described in published literature, software must be made available to editors and reviewers. We strongly encourage code deposition in a community repository (e.g. GitHub). See the Nature Research guidelines for submitting code & software for further information.

Data

Policy information about availability of data

All manuscripts must include a data availability statement. This statement should provide the following information, where applicable:

- Accession codes, unique identifiers, or web links for publicly available datasets
- A list of figures that have associated raw data
- A description of any restrictions on data availability

Short read data have been uploaded to the SRA on NCBI under PRJNA625074. Intermediate data is available on Dryad: https://doi.org/10.6078/D1B12G

Field-specific reporting

Please select the one below that is the best fit for your research. If you are not sure, read the appropriate sections before making your selection.

[x] Life sciences [ ] Behavioural & social sciences [ ] Ecological, evolutionary & environmental sciences

For a reference copy of the document with all sections, see nature.com/documents/nr-reporting-summary-flat.pdf

Life sciences study design

All studies must disclose on these points even when the disclosure is negative.

Sample size	Sample sizes reflect the number of embryos sequenced. More than 4 D. pseudoobscura samples were collected for each condition (developmental stage and sex). Because the sex of the embryos can only be determine after sequencing, the number of embryos for each sex are subject to sampling error. The number of embryos were selected based on the balance of experimental feasibility and sequencing cost.
Data exclusions	No data was excluded.
Replication	Multiple embryos for each condition (sex and devleopmental stage) were used as biological replicates. Analyses were done taking into account all replicates. The inherent noise associated with ChIP-seq data and TE insertion calls necessitates replicates being used together as opposed to comparison between individual replicates.
Randomization	Developmental staging of embryos are done by hand, therefore randomization is impossible.
Blinding	The treatment conditions for embryo collection (sex and developmental stages) are not subject to biases or placebo effects. Therefore blinding is not applicable.

Reporting for specific materials, systems and methods

We require information from authors about some types of materials, experimental systems and methods used in many studies. Here, indicate whether each material, system or method listed is relevant to your study. If you are not sure if a list item applies to your research, read the appropriate section before selecting a response.

Materials & experimental systems

n/a	Involved in the study	
[ ]	[x]	Antibodies
[x]	[ ]	Eukaryotic cell lines
[x]	[ ]	Palaeontology and archaeology
[ ]	[x]	Animals and other organisms
[x]	[ ]	Human research participants
[x]	[ ]	Clinical data
[x]	[ ]	Dual use research of concern

Methods

n/a	Involved in the study	
[ ]	[x]	ChIP-seq
[x]	[ ]	Flow cytometry
[x]	[ ]	MRI-based neuroimaging

Antibodies

Antibodies used	H3K9me3 Antibody ChIP-Seq Grade (Diagenode Catalog # C15410193)
Validation	As per Diagenode website:<br>"ChIP was performed with 1 µg of the Diagenode antibody against H3K9me3 (Cat. No. C15410193) on sheared chromatin from 1,000,000 HeLa cells using the “iDeal ChIP-seq” kit as described above. The IP'd DNA was subsequently analysed on an Illumina HiSeq 2000. Library preparation, cluster generation and sequencing were performed according to the manufacturer's instructions. The 50 bp tags were aligned to the human genome using the BWA algorithm. Figure 2A shows the signal distribution along the long arm of chromosome 19 and a zoomin to an enriched region containing several ZNF repeat genes. The arrows indicate two satellite repeat

regions which exhibit a stronger signal. Figure 2C and D show the enrichment at the KCNQ1 and H19 imprinted genes."

Animals and other organisms

Policy information about studies involving animals; ARRIVE guidelines recommended for reporting animal research

Laboratory animals	Drosophila pseudoobscura (Strain SS-R2) embryos, sex determined after sequencing.<br>Drosophila miranda (Strain MSH22) embryos, sex determined after sequencing.<br>Drosophila melanogaster (Strain Oregon-R) embryos, mixed sex pooled embryos.
Wild animals	All fly strains are from laboratory stocks. No wild animals were used in this study.
Field-collected samples	No field collected samples were used in this study.
Ethics oversight	No vertebrate animals used. No ethical approval necessary.

Note that full information on the approval of the study protocol must also be provided in the manuscript.

ChIP-seq

Data deposition

- [x] Confirm that both raw and final processed data have been deposited in a public database such as GEO.
- [x] Confirm that you have deposited or provided access to graph files (e.g. BED files) for the called peaks.

Data access links
May remain private before publication.

https://www.ncbi.nlm.nih.gov/Traces/study/?acc=PRJNA625074&o=acc_s%3Aa
https://doi.org/10.6078/D1B12G

Files in database submission

pse_stage7_M.merge.enrich
pse_stage7_F.merge.enrich
pse_stage5_M.merge.enrich
pse_stage5_F.merge.enrich
mir_sample_info.stage7.male.merge.enrich
mir_sample_info.stage7.female.merge.enrich
mir_sample_info.stage5.male.merge.enrich
mir_sample_info.stage5.female.merge.enrich
mir_sample_info.stage4early.male.merge.enrich
mir_sample_info.stage4early.female.merge.enrich
pse.RNAseq.genes.counts
normalized.RNAseq.readcounts.txt
mir.RNAseq.genes.counts
DBCC018C_S32_L006_R1_001.split1.insert
DBCC018F_S35_L006_R1_001.split1.insert
DBCC018I_S38_L006_R1_001.split1.insert
DBCC018L_S41_L006_R1_001.split1.insert
DBCC019A4_S25_L002_R1_001.split1.insert
DBCC019D4_S28_L002_R1_001.split1.insert
DBCC020C_S3_L004_R1_001.split1.insert
DBCC021O_S15_L008_R1_001.split1.insert
DBCC021R_S18_L008_R1_001.split1.insert
DBCC021U_S21_L008_R1_001.split1.insert
DBCC021X_S24_L008_R1_001.split1.insert
DBCC026A10_S8_L001_R1_001.split1.insert
DBCC026A12_S10_L001_R1_001.split1.insert
DBCC026A6_S4_L001_R1_001.split1.insert
DBCC026A8_S6_L001_R1_001.split1.insert
DBCC026C10_S40_L001_R1_001.split1.insert
DBCC026C12_S42_L001_R1_001.split1.insert
DBCC026C14_S44_L001_R1_001.split1.insert
DBCC026C16_S46_L001_R1_001.split1.insert
DBCC026C2_S32_L001_R1_001.split1.insert
DBCC026C4_S34_L001_R1_001.split1.insert
DBCC026C6_S36_L001_R1_001.split1.insert
DBCC029B_S2_L006_R1_001.split1.insert
DBCC029D_S4_L006_R1_001.split1.insert
DBCC029F_S6_L006_R1_001.split1.insert
DBCC029H_S8_L006_R1_001.split1.insert
DBCC029J_S10_L006_R1_001.split1.insert

DBCC029L_S12_L006_R1_001.split1.insert
DBCC029N_S14_L006_R1_001.split1.insert
DBCC029P_S16_L006_R1_001.split1.insert
DBCC031B_S42_L007_R1_001.split1.insert
DBCC031D_S44_L007_R1_001.split1.insert
DBCC031F_S46_L007_R1_001.split1.insert
DBCC031H_S48_L007_R1_001.split1.insert
DBCC031J_S50_L007_R1_001.split1.insert
DBCC031L_S52_L007_R1_001.split1.insert
DBCC039B10_S68_L002_R1_001.split1.insert
DBCC039B11_S69_L002_R1_001.split1.insert
DBCC039B12_S70_L002_R1_001.split1.insert
DBCC039B4_S62_L002_R1_001.split1.insert
DBCC039B5_S63_L002_R1_001.split1.insert
DBCC039B6_S64_L002_R1_001.split1.insert
mir_inputlibrary_info.txt
pAAin_a1.split1.insert
pABin_a1.split1.insert
pACin_a1.split1.insert
pADin_a1.split1.insert
pAEin_a1.split1.insert
pAFin_a1.split1.insert
pAGin_a1.split1.insert
pAHin_a1.split1.insert
pAIin_a1.split1.insert
pAJin_a1.split1.insert
pAKin_a1.split1.insert
pAMin_a1.split1.insert
pANin_a1.split1.insert
pAOin_a1.split1.insert
pAPin_a1.split1.insert
pAQin_a1.split1.insert
pARin_a1.split1.insert
pASin_a1.split1.insert
pATin_a1.split1.insert
pAUin_a1.split1.insert
pAVin_a1.split1.insert
pAWin_a1.split1.insert
pAXin_a1.split1.insert
pAin_a1.split1.insert
pBin_a1.split1.insert
pCin_a1.split1.insert
pDin_a1.split1.insert
pEin_a1.split1.insert
pFin_a1.split1.insert
pGin_a1.split1.insert
pHin_a1.split1.insert
pMin_a1.split1.insert
pNin_a1.split1.insert
pOin_a1.split1.insert
pTin_a1.split1.insert
pWin_a1.split1.insert
pXin_a1.split1.insert
pYin_a1.split1.insert
pZin_a1.split1.insert
pse_inputlibrary_info.txt

Genome browser session
(e.g. UCSC)

NA

Methodology

Replicates

See Supplementary table 2

Sequencing depth

See Supplementary table 2

Antibodies

H3K9me3 Antibody ChIP-Seq Grade (Diagenode Catalog # C15410193)

Peak calling parameters	No peak calling was used.
Data quality	No peak calling was used.
Software	bwa (r0.7.15)<br>bowtie2 (v2.3.0)<br>bedtools (v2.26.0)<br>samtools (v1.5)<br>RepeatMasker (v3.3)<br>featureCounts (v1.6.2, part of subread package)<br>Rstudio (v1.3)<br>IRanges (v2.22)<br>DEseq2 (v1.28.1)
