# Supplementary material for: Epigenetic conflict on a degenerating Y chromosome increases mutational burden in Drosophila males
Source: Nat Commun. 2020 Nov 2;11:5537. doi: 10.1038/s41467-020-19134-9 (PMC7608633; doi:10.1038/s41467-020-19134-9)
Supplement: Supplementary file 4 — Description of Additional Supplementary Files [file 41467_2020_19134_MOESM4_ESM.pdf]

**Description of Additional Supplementary Files**

File name: Supplementary Data 1.

DNA-seq and RNA-seq summary

File name: Supplementary Data 2.

ChIP-seq library and mapping summary

File name: Supplementary Data 3.

de novo TE insertion identification for each library

File name: Supplementary Data 4.

*"de novo"* gene insertion identification for each library
