# Supplementary material for: Epigenetic conflict on a degenerating Y chromosome increases mutational burden in Drosophila males
Source: Nat Commun. 2020 Nov 2;11:5537. doi: 10.1038/s41467-020-19134-9 (PMC7608633; doi:10.1038/s41467-020-19134-9)
Supplement: Supplementary file 2 — Peer Review [file 41467_2020_19134_MOESM2_ESM.pdf]

Reviewer #1 (Remarks to the Author):

This is a very good paper. It has long been known that transposable elements (TEs) accumulate on the nonrecombining portions of Y chromosomes. The novelty of this paper is to show that a young Y chromosome which still has many functional genes is a hotbed of active TEs, apparently because it cannot be transcriptionally silenced. New insertions of TEs lead to further degradation of the Y and also the rest of the genome.

All of the following comments are minor and can easily be addressed in revision:

The paper talks in several spots about how the young "toxic" Y chromosome imposes a mutational load on males. But if I'm interpreting Fig. 6 correctly, the D. miranda Y is the source of many additional TE insertions on the autosomes, which of course degrades the fitness of both sexes.

p. 5, end of full para: The finding that TEs on the Y that lie closer to expressed genes is to me one of the paper's most interesting results. I suggest putting this point near the beginning of the paragraph or in some other way giving it more visibility. It is buried here.

p. 7, l. 10: "The accumulation of repetitive elements on the Y chromosome appears to be universal...." Common, but not universal. Some Ys show no degeneration -- the fugu Y is a famous example.

Fig. 3: Panels C – F: Show which male-female contrasts are significantly different. Panels G, H: I found these hard to understand, and am not clear that they add anything to Panels C – F. Caption: "the vertical line denotes whether...." Which vertical line?

Fig. 4, Panels G – H: Are the points shown binned from the original data? If not, one would expect many more points, and it would be hard to believe the p values that are given.

Fig. 5: All panels: The ordering of A-B-C-D-E-F is strange and confusing. Panels C – D: Consider plotting the Y axes on the same scale, which would make much clearer how different pseudo and miranda are. Panel E: See query about binning in Fig. 4. Panel E and caption: If these are referring to the standard Pearson correlation coefficient, please write it as r². Panel F: Again, consider making the scale of the Y axes of the two histograms the same.

Reviewer #2 (Remarks to the Author):

This study by Wei and colleagues describes transposable element expression in two species of Drosophila. While potentially interesting a much more detailed analysis is needed in order to evaluate the outcomes and the data interpretation in this study. The results are bit scattered and not sufficiently detailed in my opinion. I also have concerns about data interpretation and language (a few examples are given below).

1. This title is highly problematic. "Epigenetic conflict creates toxic Y chromosome and increased mutational burden in Drosophila males" There is no experimental evidence of epigenetic conflict, there is no evidence of a "toxic" Y chromosome, and the evidence of a mutational burden in males is rather underwhelming and not that clear.

2. The second half of the first sentence of the abstract is also problematic: "the establishment of transcription repressing heterochromatin during early development safeguards genome integrity". This is not correct for "eukaryotes" in general as the first half of the sentence would have you believe. It is not correct for mammals either. I believe that the authors are talking about a case in Drosophila.

3. The authors state that "TEs may exploit vulnerabilities associated with incomplete genomic defenses". TEs do not "exploit vulnerabilities".

4. Similar issue with wording here: "epigenetically naïve". etc etc etc.

5. The authors conclude that there is evidence for an "epigenetic conflict on evolving Y chromosomes between transcription of essential genes and silencing of selfish DNA." It is entirely unclear what this evidence is.

6. "male--biased TE misexpression diminishes across development in D. pseudoobscura, but remains elevated in D. miranda, the species with the younger and larger Y chromosome." While it is hard for me to envision how the results could have been much different than this given that D. miranda Y is full of expressed genes and also larger. Yet the reduction in male bias TE expression that is suggested to occur in D. pseudoobscura is hardly strong relative to what is observed in D. miranda, and frankly hardly diminishes across development. The evidence here is rather underwhelming indeed.

7. Figure 1b. These profiles should not be presented in fig 1 without a citation and in reference to specific datasets.

8. "normalized read count" is not clear. Please use standard language and metrics for analysis of expression. As presented, it creates confusion in the interpretation too.

9. Fig 3 is underwhelming. There are hardly any difference between D. pseudoobscura and D. miranda.

10. Fig 5a and 5b have issues. One: The x-axis scale in fig 5a and b is vastly different, this can cause serious artifacts Two: there are some major outlier in figure 5a and 5b. Three: later states do not show a greater number of insertions as should be expected. None of this is even mentioned in the results or discussed at all.

11. Fig 5cd: the interpretation of the residuals is not clear.

12. Figure 5f: the higher y-axis scale in male is driven by insertions into the Y chromosome. A few issues come to mind. First, ascertainment of male insertions are particularly uncertain due to the issues with the assembly of the Y chromosome. Second, even if this greater number of insertions on the Y were true (which I don't think it is), one would still have to explain why the Y chromosome has these new insertions, in contrast to the other chromosomes that seem to be very similar in males and females. Third, chromosome AD has a disproportional number of insertions in females, which goes contrary to the explanation put forward by the authors.

13. Overall, I think that the authors need to provide a lot more details about fig 5. Also, there needs to be careful experimental validation of the insertions. I am not at all convinced that these insertions are not artifactual.

14. Mapping and analysis of TE reads are notoriously fraught with problems. The authors did little to show that any of their results is robust to alternative mapping strategies. It is also unclear if issue with the assembly of the Y chromosome in these two species played a role.

Reviewer #3 (Remarks to the Author):

The article by Wei et al examines the methylation patterns associated with TEs on a neo-Y chromosome. They show that TEs on the Y-chromosome likely piggy-back on expression of gene

sequences to evade silencing. They are associated with ongoing copying events. The analysis of embryos in D. miranda and D. pseudoobscura.

I have only one major technical concern and one major concern regarding interpretation:
TEs are inherently repetitive and it can be difficult to distinguish copies. I did not see a discussion in the text of how or whether this was handled to ensure that copies on the Y are distinguished from similar copies on the autosomes. This needs to be discussed in the text. Similarly, split read mappings can be prone to mis-mapping. Are there overhang criteria that were used to exclude mis mapped reads? It seems fairly important given the current study's goals.

The authors conclude that epigenetic arms races drive a toxic Y chromosome. I think it important to distinguish the (likely detrimental) effects of TE proliferation from a cytotoxic killer Y chromosome. If there are other reasons to believe these neo-Y chromosomes are indeed toxic rather than a mutational burden, then the evidence needs to be explained fully and cited. If it is merely mutational, then I think a more nuanced discussion of how these are expected to shape future evolution would be warranted.

The fact that it does not kill cells may diminish the impact slightly compared with the title, but I would imagine this work will be highly cited by the community working in sex chromosome evolution as well as Drosophila biologists. Any Nature journal would be lucky to have this paper if the above issues can be handled appropriately.

<u>Response to Reviewer #1</u> (Remarks to the Author):

This is a very good paper. It has long been known that transposable elements (TEs) accumulate on the nonrecombining portions of Y chromosomes. The novelty of this paper is to show that a young Y chromosome which still has many functional genes is a hotbed of active TEs, apparently because it cannot be transcriptionally silenced. New insertions of TEs lead to further degradation of the Y and also the rest of the genome.

All of the following comments are minor and can easily be addressed in revision:

The paper talks in several spots about how the young "toxic" Y chromosome imposes a mutational load on males. But if I'm interpreting Fig. 6 correctly, the D. miranda Y is the source of many additional TE insertions on the autosomes, which of course degrades the fitness of both sexes.

This is a good point, and we state that now more explicitly in the paper on p. 7. The 'toxic Y' creates a direct mutational burden in males, and additional TE insertions on the X and autosome can degrade fitness in both sexes. Intriguingly, the female genome is larger in *D. miranda* than *D. pseudoobscura* (both based on flow cytometry and genome assemblies), and this difference can largely be attributed to an accumulation of TEs. In addition, although we cannot distinguish between germline vs somatic insertions given our strategy, a large proportion of the insertions are likely somatic (since there are at least 3 orders of magnitudes more somatic cells than germ/pole cells at this embryonic stage) and would not propagate to the next generation. The fitness impact and deleterious potential of these somatic insertions (as result of poor suppression of TEs on the neo-Y) are therefore restricted to males.

p. 5, end of full para: The finding that TEs on the Y that lie closer to expressed genes is to me one of the paper's most interesting results. I suggest putting this point near the beginning of the paragraph or in some other way giving it more visibility. It is buried here.

We modified the paragraph, and put this observation as a hypothesis on the beginning of the paragraph.

p. 7, l. 10: "The accumulation of repetitive elements on the Y chromosome appears to be universal...." Common, but not universal. Some Ys show no degeneration -- the fugu Y is a famous example.

Modified.

Fig. 3: Panels C – F: Show which male-female contrasts are significantly different. Panels G, H: I found these hard to understand, and am not clear that they add anything to Panels C – F. Caption: "the vertical line denotes whether...." Which vertical line?

Panels C and F show that individual TEs (as opposed to aggregates in the boxplots of B and D) become less male-biased in *D. pseudoobscura* and more male-biased in *D. miranda*. The dots and arrowheads (connected by vertical lines) represent the fold expression difference between sex for each TE at developmental stage 5 (dots) and stage 12 (arrowheads), and the color of the line indicates whether the fold change is becoming more (blue) or less (red) male-biased. We modified the Figure legend to make this point more clearly.

Fig. 4, Panels G – H: Are the points shown binned from the original data? If not, one would expect many more points, and it would be hard to believe the p values that are given.

These panels use only a subset (n=198) of the repeats plotted in figures 2 through 3 (n=303). This is because many of the TE entries in the repeat index show no masking/annotation by RepeatMasker on the neo-Y (likely because they are *D. pseudoobscura* specific, or not found on the neo-Y contigs in *D. miranda*). We state this now clearly in the legend. Previously, we erroneously labeled the R-squared as r (perason's correlation); this is now corrected. The p-value is inferred from the linear regression and is correct. We also changed Figure 4H which previously showed the fold-difference between male and female stage 5 embryos, to that of stage 12 embryos to keep it consistent with Figure 4G.

Fig. 5: All panels: The ordering of A-B-C-D-E-F is strange and confusing. Panels C – D: Consider plotting the Y axes on the same scale, which would make much clearer how different pseudo and miranda are. Panel E:

See query about binning in Fig. 4. Panel E and caption: If these are referring to the standard Pearson correlation coefficient, please write it as r^2. Panel F: Again, consider making the scale of the Y axes of the two histograms the same.

We have rearranged the panels. Separate linear regressions were used for the *D. pseudoobscura* and *D. miranda* insertion counts. Therefore, the residuals are not directly comparable and plotting them at the same scale may be misleading. In these analyses, the main comparison is between sexes and stages of the same species. Regarding Panel E (now panel G), only TEs where we find evidence of unique insertions are plotted, and therefore represents a subset of all TEs in the repeat index. We are using the R-squared here not Pearson's correlation coefficient. This is now all stated more clearly in the manuscript.

<u>Response to Reviewer #2</u> (Remarks to the Author):

This study by Wei and colleagues describes transposable element expression in two species of Drosophila. While potentially interesting a much more detailed analysis is needed in order to evaluate the outcomes and the data interpretation in this study. The results are bit scattered and not sufficiently detailed in my opinion. I also have concerns about data interpretation and language (a few examples are given below).

1. This title is highly problematic. "Epigenetic conflict creates toxic Y chromosome and increased mutational burden in Drosophila males" There is no experimental evidence of epigenetic conflict, there is no evidence of a "toxic" Y chromosome, and the evidence of a mutational burden in males is rather underwhelming and not that clear.

We modified our title to "Epigenetic conflict on a degenerating Y chromosome increases mutational burden in Drosophila males". The reviewer is correct that we don't experimentally demonstrate an epigenetic conflict but rather infer it from the observation that transcription of zygotic neo-Y genes is associated with regions of reduced heterochromatin formation; 'toxic' Y chromosome is a term used in the literature (i.e. Marais et al. 2018) to describe the effect that repetitive elements on the Y may be insufficiently repressed, which we show in Figs. 4B-H (but that phrase has been removed from the title). Reduced H3K9me3 enrichment is associated with higher numbers of TE insertions in males (i.e. a mutational burden). Please see our replies to points 10-13 demonstrating that our findings of elevated TE insertion rates in male *D. miranda* is robust. We have added multiple analyses, intermediate data, and figure panels to clarify and support our approach and results. Further, we have also now mentioned in text that the higher insertion rate in males is also consistent with the female biased sex ratio that has been documented by Dobzhansky, and shorter lifespan of *D. miranda* males.

2. The second half of the first sentence of the abstract is also problematic: "the establishment of transcription -repressing heterochromatin during early development safeguards genome integrity". This is not correct for "eukaryotes" in general as the first half of the sentence would have you believe. It is not correct for mammals either. I believe that the authors are talking about a case in Drosophila.

We add "in Drosophila" to this sentence to clarify.

3. The authors state that "TEs may exploit vulnerabilities associated with incomplete genomic defenses". TEs do not "exploit vulnerabilities".

We modify this sentence to "…*and incomplete epigenomic defenses during early development may lead to TE mobilization*".

4. Similar issue with wording here: "epigenetically naïve". etc etc etc.

"Epigenetic naïve" is a term used in the scientific literature to i.e. Li et al. eLife 2014, and we modified the sentence to define this term: "*In most animals, the zygotic genome is initially inactive and epigenetically 'naïve' (i.e. it contains few chromatin features)* [1],…."

5. The authors conclude that there is evidence for an "epigenetic conflict on evolving Y chromosomes between transcription of essential genes and silencing of selfish DNA." It is entirely unclear what this evidence is.

The evidence for this statement can be found in Figure 4. We show that segments of the neo-Y adjacent to zygotically active genes are less heterochromatic, and TEs near Y-linked zygotically expressed genes are less enriched for heterochromatin than TEs across the entire chromosome. Consistently, TEs are expressed more highly in males if they have a larger number of insertions around zygotically expressed neo-Y genes, and these TEs show higher male-bias in their expression. This is reminiscent of a similar pattern with (euchromatic) TEs inserted near genes in *D. melanogaster*: Because of epigenetic silencing and spreading of H3K9me3 into neighboring genes, these TE insertions are under negative selection (Lee 2015). This is now discussed in the main text of the manuscript. In *D. miranda*, the mix of repeats and genes on the neo-Y creates a conflict between the need to epigenetically silence the former while permitting the expression of the latter.

6. "male---biased TE misexpression diminishes across development in D. pseudoobscura, but remains elevated in D. miranda, the species with the younger and larger Y chromosome." While it is hard for me to envision how the results could have been much different than this given that D. miranda Y is full of expressed genes and also larger. Yet the reduction in male bias TE expression that is suggested to occur in D. pseudoobscura is hardly strong relative to what is observed in D. miranda, and frankly hardly diminishes across development. The evidence here is rather underwhelming indeed.

We actually expected that neo-Y TEs would eventually become more down-regulated, as heterochromatin establishment progressed during development. Thus, to us it was surprising that neo-Y TEs remained highly expressed once heterochromatin is fully formed in later developmental stages. Figures 2C (pseudoobscura) vs. 2D (miranda) clearly show that male-biased expression of repeats diminishes during development in *D. pseudoobscura* but not so in *D. miranda*. (We have now added astericks to mark significance in male-biased expression above boxes to make the contrast more obvious). Specifically, in D. pseudoobscura, males have on average 1.68-fold higher TE expression than females at stage 5 and 1.26-fold higher at stage 10, 1.00-fold higher at stage 12, while in D. miranda males have on average 1.67-fold higher TE expression than females at stage 5, 2.47-fold higher at stage 10, and 2.05-fold higher at stage 12. This is further exaggerated when we remove the lowly expression TEs (normalized read counts < 4 across all samples): stages 5, 10, and 12 are 1.77, 1.31, and 1.01-fold higher in *D. pseudoobscura* but 1.87, 2.70, and 2.29-fold higher in *D. miranda*.

7. Figure 1b. These profiles should not be presented in fig 1 without a citation and in reference to specific datasets.

We include five citations that include datasets containing transcription profiles during embryonic development and formation of heterochromatin that were used to generate this schematic representation of transcriptional changes and heterochromatin regulation during early embryonic development (ref. 15-19) when first referring to this figure in the text.

8. "normalized read count" is not clear. Please use standard language and metrics for analysis of expression. As presented, it creates confusion in the interpretation too.

As far as we know read count is not an obscure metric and is in fact the preferred input for ubiquitously used differential expression programs such as DESeq and EdgeR both of which have been cited thousands of times. (DEseq2: Love, Huber & Anders 2014 currently has 17329 citations). As mentioned in the materials and methods, the RNA-seq counts are normalized by the counts of autosomal genes using DEseq2's pipeline; this is under the assumption that autosomal genes are more-or-less equally expressed between the sexes and samples. And the DNA-seq counts are normalized (divided) by the average autosomal coverage of the sample which provides the number of reads mapped to TE per 1x coverage.
Metrics like RPKM and FPKM are actually not ideal for comparing between sexes because they do not take into account of the fact that the male and female genomes are not the same size and, specifically for D. miranda, have very different numbers of genes. Nevertheless, we have included the RNAseq results with RPKM in Supplementary figure S2. The same male bias is also observed.

9. Fig 3 is underwhelming. There are hardly any difference between D. pseudoobscura and D. miranda.

Indeed, as the title of the figure indicates ("Y-linked TEs drive male-biased expression in <u>both</u> *D. pseudoobscura* and *D. miranda*"), Figure 3 highlights important commonalities between *D. pseudoobscura*

and *D. miranda*, but it also reveals interesting differences that are discussed in the manuscript. In particular, TEs located on the Y chromosome appear to drive most of the male-biased expression of TEs in both species (Fig. 3C-3F). However, there are substantially more Y-biased TEs in *D. miranda* than in D. pseudoobscura (20 vs. 79; 3A vs. 3B). Moreover, while male-biased expression decreases for most TEs during development in *D. pseudoobscura* (Fig 3G; also 3C and 3E), the opposite is true for *D. miranda* (Fig 3H; also 3D and 3F).

10. Fig 5a and 5b have issues. One: The x-axis scale in fig 5a and b is vastly different, this can cause serious artifacts Two: there are some major outlier in figure 5a and 5b. Three: later states do not show a greater number of insertions as should be expected. None of this is even mentioned in the results or discussed at all.

Regarding one: We assume that the reviewer meant Fig. 5A and 5C (now C and E), as A and B are different plots and therefore have completely different X-axes. Female and male samples of the two species are sequenced to different coverage and thus have different x-axis. The key point of these analyses is the differences between sexes of the *same* species. Re two: We have redone the analysis after removing the outlier points (shown in supplementary figure S7). The same pattern (i.e. higher number insertions in *D. miranda* males) holds. Re: three: Later stages do indeed have significantly more insertions (ANOVA of the "stage" variable), as expected from having more cells – this is mentioned in the main text.

11. Fig 5cd: the interpretation of the residuals is not clear.

To remove the effect of autosomal coverage (library size), for each species we generated a linear regression with autosomal coverage as the independent variable and the number of unique insertions as the dependent variable. This regression and the R-squared is capturing the amount variance in the number of insertions as a result of variance in the library size. The residuals of the samples are therefore capturing the remaining variance when the effect of library size is removed. The variance in the residuals is then contributed by the remaining (sex and stage) independent variables, potentially unidentified variables, and noise. To show the effect of sex and stage, we the grouped the samples accordingly for the boxplots. Therefore, the residuals plotted represent how the number of unique insertions differ across sex and developmental stage after accounting for the contribution of library size.

12. Figure 5f: the higher y-axis scale in male is driven by insertions into the Y chromosome. A few issues come to mind. First, ascertainment of male insertions are particularly uncertain due to the issues with the assembly of the Y chromosome. Second, even if this greater number of insertions on the Y were true (which I don't think it is), one would still have to explain why the Y chromosome has these new insertions, in contrast to the other chromosomes that seem to be very similar in males and females. Third, chromosome AD has a disproportional number of insertions in females, which goes contrary to the explanation put forward by the authors.

More TE insertions in males is not solely driven by insertions on the Y. Importantly, Figure S8 shows that male *D. miranda* show more TE insertions, even when only considering only autosomes. Thus, even if we were to ignore the Y, we still would infer more TE insertions in males than females. Re point 1, it is not clear to us how assembly mistakes would result in wrongly inferring *de novo* TE insertions in a particular embryo. Reads from all embryos are mapped to the same assembly, and if an assembly mistake would result in incorrectly calling a TE insertion, this insertion would be shared among all embryos and not counted as a *de novo* insertion, as we require insertions to be found in only one sample (see Methods for details). Re point 2, we can only speculate why this may be the case (we mention transposition bias or selection as possible contributors to the non-random spatial distribution of TEs in our paper), and identifying the mechanism for insertional bias of TEs is not relevant for our point, and beyond the scope of the paper. Re point 3, males are hemizygous for the X (Muller AD), thus fewer copies of TE insertions may in fact be expected in males that have only a single X relative to females (i.e. the X provides a smaller mutational target in males).

13. Overall, I think that the authors need to provide a lot more details about fig 5. Also, there needs to be careful experimental validation of the insertions. I am not at all convinced that these insertions are not artifactual.

To provide more details of the analyses, we now included schematics of the pipeline and rationale (A and B) of the experiment in Fig 5. We have further included in supplementary table S3 and S4 documenting the

extent of our filtering and analyses strategies to distinguish between artifacts (like chimeric reads) and real insertions.

Importantly, we were very cautious and extremely conservative to infer novel TE insertions. Most studies that use sequencing-based approaches to infer novel TE insertions only require identification of one junction of the TE insertion. This could indeed create artifactual inferences of novel TE insertions due to chimeric sequencing reads (a rare but not impossible event). However, we require identification of both junctions of a putative TE insertions, which is highly unlike to result from chimeric reads. We verified the stringency of our analysis pipeline by mapping sequencing reads to exonic sequences (which are not expected to move around in the genome, but may produce chimeric reads) instead of the TE index. The genomic sequence was masked by both the TE index and the exonic sequences, and junction and insertion identification was done as according to Figure 5A and B. As shown in Supplementary Table S4, this procedure resulted in the detection of only a handful of "exon insertions"; the median number of novel "exon insertions" was 0 across libraries (and the maximum number was 4). This demonstrates that our pipeline is very effective in eliminating chimeric reads, and validates that our methodology for inferring *de novo* TE insertions is very conservative.

14. Mapping and analysis of TE reads are notoriously fraught with problems. The authors did little to show that any of their results is robust to alternative mapping strategies. It is also unclear if issue with the assembly of the Y chromosome in these two species played a role.

TE transcript abundance and copy number analyses in Figure 2 and 3 are inferred from mapping to a repeat index instead of the whole genome (and thus not influence by assemblies). Demonstrating that male-biased TE expression is not an artifact of mapping strategy, we are including in Supp figure 2 a heatmap of the TE transcript abundance using bowtie2; it clearly illustrates the same patterns a figure 2A, and Suppl. Fig. 9 shows that mapping with bowtie2 is very similar to BWA. For the ChIP-seq analyses, we are including the H3K9me3 enrichment profiles when we use only uniquely mapping reads (Supplementary figure S5). We similarly find that the bulk of the neo-Y is not as enriched as pericentromeric heterochromatin.
Regarding the insertion analyses, the calls relied on unique mappings of one mate pair to the repeat-masked genome with the other mate pair mapped to the repeat index (the two pairs are separately mapped as single ends, see figure 5A). Thus, we show that our results are robust with regards to mapping strategies, and are not relying on fraught and nonunique mapping of repetitive sequences to the genome.

<u>Response to Reviewer #3</u> (Remarks to the Author):

The article by Wei et al examines the methylation patterns associated with TEs on a neo-Y chromosome. They show that TEs on the Y-chromosome likely piggy-back on expression of gene sequences to evade silencing. They are associated with ongoing copying events. The analysis of embryos in D. miranda and D. pseudoobscura.

I have only one major technical concern and one major concern regarding interpretation:
TEs are inherently repetitive and it can be difficult to distinguish copies. I did not see a discussion in the text of how or whether this was handled to ensure that copies on the Y are distinguished from similar copies on the autosomes. This needs to be discussed in the text. Similarly, split read mappings can be prone to mis-mapping. Are there overhang criteria that were used to exclude mis mapped reads? It seems fairly important given the current study's goals.

This is an inherent issue of studying repeats - i.e. we cannot identify the exact origin of reads and transcripts. For any given TE family, transcripts originating from insertions on autosomes are mostly indistinguishable from Y-linked insertions, especially for highly prolific, young TEs. However, we can infer whether a specific TE family is enriched on the Y chromosome, based on genomic read coverage patterns. TE families that are not particularly enriched on the Y chromosome will have similar read coverage in male and females, while repeats that are enriched on the Y will show higher read coverage in males relative to females. We discuss this at the end of page 3 / beginning of page 4. We have multiple lines of evidence consistent with elevated expression from the Y-linked copies causing male-biased expression, including Y-enriched TEs showing more male biased expression, and reduced H3K9me3 enrichment around Y-linked TEs, particularly those near Y-linked genes.

Regarding the ChIP-seq analyses, H3Kme3 enrichment was inferred in part from non-unique mapping of TEs to the genome (BWA by default randomly assigns a read to a target if there are multiple equally good mappings). We have now included Supplementary figure S5 showing that even if we use unique mapping, we see the same pattern of reduced H3K9me3 enrichment on the neo-Y of *D. miranda* as compared to pericentromeric heterochromatin.

Regarding split read mapping (which we understand to mean a single read has split mapping to different places, e.g. half of the read to a unique region and the other half to a repeat), we do not use this mapping strategy. For figure 2 and 3, the read counts (for transcript abundance and copy number approximation) are exclusively done using pair-end mappings to the repeat index. For the unique insertion analyses, we separately mapped the read pairs as single ends and looked for pairs where one read maps to an entry on the repeat index and the other maps to a unique region in the repeat-masked genome. Split-mapped reads are not considered.

The authors conclude that epigenetic arms races drive a toxic Y chromosome. I think it important to distinguish the (likely detrimental) effects of TE proliferation from a cytotoxic killer Y chromosome. If there are other reasons to believe these neo-Y chromosomes are indeed toxic rather than a mutational burden, then the evidence needs to be explained fully and cited. If it is merely mutational, then I think a more nuanced discussion of how these are expected to shape future evolution would be warranted.

Here, the ‘toxic Y’ specifically refers to the mutational burden and deleterious potential due to misregulated TE activity on the degenerating Y (a term that is used in the evolutionary literature). We have elaborated and clarified on this point in the text, and put this term in quotation marks to make this clear.

The fact that it does not kill cells may diminish the impact slightly compared with the title, but I would imagine this work will be highly cited by the community working in sex chromosome evolution as well as Drosophila biologists. Any Nature journal would be lucky to have this paper if the above issues can be handled appropriately.

Thank you for the comment. We unfortunately do not have direct evidence that cells or male flies are dying due to elevated TE activities and *de novo* insertions (although there is a female-biased sex ratio in this species which is consistent with reduced male fitness, and males live shorter than females in D. miranda), and this is now mentioned in the text. However, TE activities are well-appreciated as a source of deleterious impact on organismal fitness across many contexts, including fertility, genome integrity, cancer, and aging.

Reviewer #1 (Remarks to the Author):

The revisions have made a strong ms even stronger. Several of the additions, e.g. on the effects of somatic TE insertions (p. 7), are very helpful. By necessity because of the nature of repetitive elements and the novel methods developed here, this a complicated paper that's a bit of a challenge for the reader. But I have no reservations about recommending it for publication. The following suggestions are all trivial.

Abstract: "... of different ages."
Abstract: "male-biased TE misexpression..." I'm unclear what TE misexpression means.
p. 3: "TEs are activated..." Does "activated" mean transcribed, or excised, or something else?
p. 5: "windows overlapping annotated genes have significantly lower...." Say how much lower.
p. 6: "deposition and spreading of silencing heterochromatin at TEs near euchromatic genes is deleterious...."
References: Lots of typos here.
Fig 1: I have a hard time understanding the karyotypes. In subobscura, is the longer chromosome at 2 o'clock Muller A? The caption might mention that in pseudo the ancestral Y was lost. In pseudo and miranda, give the Muller element names for the neo-Ys.

Reviewer #3 (Remarks to the Author):

The authors have addressed my concerns appropriately, especially the questions about mapping of repetitive elements. Thanks for changing the title.

Response to Reviewer #1 (Remarks to the Author):
The revisions have made a strong ms even stronger. Several of the additions, e.g. on the effects of somatic TE insertions (p. 7), are very helpful. By necessity because of the nature of repetitive elements and the novel methods developed here, this a complicated paper that's a bit of a challenge for the reader. But I have no reservations about recommending it for publication. The following suggestions are all trivial.
Thanks!
Abstract: "... of different ages." corrected
Abstract: "male-biased TE misexpression..." I'm unclear what TE misexpression means. changed
p. 3: "TEs are activated..." Does "activated" mean transcribed, or excised, or something else? It means both, transcribed (Fig 2, 3) and mobilized (Fig 5), and thus we chose "activated" to stay agnostic.
p. 5: "windows overlapping annotated genes have significantly lower...." Say how much lower. done
p. 6: "deposition and spreading of silencing heterochromatin at TEs near euchromatic genes is deleterious...."
References: Lots of typos here. corrected
Fig 1: I have a hard time understanding the karyotypes. In subobscura, is the longer chromosome at 2 o'clock Muller A? The caption might mention that in pseudo the ancestral Y was lost. In pseudo and miranda, give the Muller element names for the neo-Ys. We modified our figure to make this more clear, and put labels on the chromosomes.

Reviewer #3 (Remarks to the Author):
The authors have addressed my concerns appropriately, especially the questions about mapping of repetitive elements. Thanks for changing the title.
Thanks!
